# Insights from the Cold Transcriptome and Metabolome of *Dendrobium officinale*: Global Reprogramming of Metabolic and Gene Regulation Networks during Cold Acclimation

**DOI:** 10.3389/fpls.2016.01653

**Published:** 2016-11-08

**Authors:** Zhi-Gang Wu, Wu Jiang, Song-Lin Chen, Nitin Mantri, Zheng-Ming Tao, Cheng-Xi Jiang

**Affiliations:** ^1^Zhejiang Institute of Subtropical Crops, Zhejiang Academy of Agricultural SciencesWenzhou, China; ^2^School of Applied Sciences, Health Innovations Research Institute, Royal Melbourne Institute of Technology University, MelbourneVIC, Australia

**Keywords:** Cold acclimation, *Dendrobium officinale*, metabolic network, regulatory mechanism, signal transduction, transcriptome

## Abstract

Plant cold acclimation (CA) is a genetically complex phenomenon involving gene regulation and expression. Little is known about the cascading pattern of gene regulatroy network and the link between genes and metabolites during CA. *Dendrobium officinale* (DOKM) is an important medicinal and ornamental plant and hypersensitive to low temperature. Here, we used the large scale metabolomic and transcriptomic technologies to reveal the response to CA in DOKM seedlings based on the physiological profile analyses. Lowering temperature from 4 to –2°C resulted in significant increase (*P* < 0.01) in antioxidant activities and electrolyte leakage (EL) during 24 h. The fitness CA piont of 0°C and control (20°C) during 20 h were firstly obtained according to physiological analyses. Subsequently, massive transcriptome and metabolome reprogramming occurred during CA. The gene to metabolite network demonstrated that the CA associated processes are highly energy demanding through activating hydrolysis of sugars, amino acids catabolism and citrate cycle. The expression levels of 2,767 genes were significantly affected by CA, including 153-fold upregulation of CBF transcription factor, 56-fold upregulation of MAPKKK16 protein kinase. Moreover, the gene interaction and regulation network analysis revealed that the CA as an active process, was regulated at the transcriptional, post-transcriptional, translational and post-translational levels. Our findings highligted a comprehensive regulatory mechanism including cold signal transduction, transcriptional regulation, and gene expression, which contributes a deeper understanding of the highly complex regulatory program during CA in DOKM. Some marker genes identified in DOKM seedlings will allow us to understand the role of each individual during CA by further functional analyses.

## Introduction

Plants frequently encounter unfavorable growth conditions that jeoparadize its growth and affect productivity. Cold is a major stress that adversely affects plant growth and limits its geographical distribution and agricultural productivity (Boyer, 1982). *Dendrobium officinale* Kimura and Migo (DOKM), belonging to *Dendrobium* genus, is an important Chinese herb that has both ornamental value and a broad range of therapeutic effects in many Asian countries (Bulpitt et al., 2007). The specie is mainly distributed in subtropical and temperate Asian and North-Australia and exhibits diverse ecological habitats (Lavarack et al., 2000; Ng et al., 2012). Under nature condition, the specie is extremely hypersensitive to low temperature above the freezing point, thereby resulting in major yeild losses (Lavarack et al., 2000). Due to extensive demand of DOKM materials in health care, DOKM has been exploited to near extinction and is now defined as an endangered medicinal plant in China (Ng et al., 2012). In practice, DOKM is commonly produced in green house for saving from cold ambient environment. It is well known that many temperate herbaceous plants in nature, have evolved adaptive mechanisms to survive seasonal low winter temperature or sudden chilling (<10°C) through a process termed cold acclimation (CA) (Guy, 1990). CA is defined as the process leading to increased cold tolerance in response to a period of exposure to low non-freezing temperatures (0–5°C) (Thomashow, 1999). Therefore, uncoveing CA molecular mechanism is essetinal in DOKM, not only for breeding elite cultivars of cold tolerance but also for lifting productivity.

Many studies have shown that CA and cold tolerance are genetically complex, quantitative traits, that are tightly associated with gene expression, enzyme activities, and the concentrations of primary and secondary metabolites (Zhu et al., 2007; Theocharis et al., 2012). For instance, several works suggested that cold triggers a highly complex regulatory program that results in extensive reorganization of the transcriptome (Oono et al., 2006; Chinnusamy et al., 2007; Hincha et al., 2012). These changes involve up- and down-regualtion of hundreds of genes occurring in waves with time of incubating plants at low temperature. Numerous cold-responsive genes (CORs) have been identified from different plant species. These genes encode a diverse array of proteins with known functions such as detoxification of reactive oxygen species (ROS), biosynthesis of carbohydrates and lipids; antifreeze proteins, late embryogenesis abundant (LEA) proteins, heat-shock proteins (HSPs); transcriptional activators related to C-repeat/dehydration-responsive element binding factors (CBFs/DREBs) (Buskirk and Thomashow, 2006; Knight and Knight, 2012; Zhao et al., 2015). In particular, a deeper understanding of COR gene regulation has been considered to be ICE1-CBF-COR module in eliciting cold tolerance (Chinnusamy et al., 2003; Thomashow, 2010). Despite considerable efforts, the multigenic CA traits cannot be explained intuitively based on changes in expression of single or a few genes. Much remains to be elucidated in the cascading pattern of gene networking and the interactions between genes and metabolites.

In addition to their complex gene networks, plants possess multiple highy regulated metabolic networks that play central regulatory roles in their growth under cold stress. It is well documated that CA leading to enhanced cold tolerance involves coordinated activation of many biochemical pathways (Usadel et al., 2008; Janská et al., 2010; Dauwe et al., 2012). For example, the glycolysis and synthesis of amino acids have gained considerable interests not only because sugars and amino acids are vital for the synthesis of functional proteins but also because they sever as precursors for a large array of metabolites with multiple functions in the response to CA (Kaplan et al., 2007; Savouré and Szabados, 2010; Krasensky and Jonak, 2012). Unfortunately, limited metabolite profiling studies have been conducted to uncover dynamic envet that can potentially capture the link between gene and metabolite networks during CA.

Despite CA complexity mentioned above, recent technical advances in genetic analysis tools, gene expression profiling, and metabolomics have made it possible to dissect the complex processes involved in plant CA (Jozefczuk et al., 2010; Hincha et al., 2012; Sinha et al., 2015). Transcriptome- and metabolome-wide approaches can allow visualization of the underlying biological processes of cold stress, leading to a better insight into the behavior responses to CA. Such research on plant CA using multiple omic tools can discover useful genes for molecular breeding and facilitate the improvement of corp yields under cold ambient environment. Previous studies on DOKM were focused on exploring molecular markers and investigating alkaloid or polysaccharide biosynthetic genes (Lu et al., 2012; Guo et al., 2013; Zhang et al., 2016). According to our knowledge, no efforts have been made to investigate the molecular basis of CA for DOKM using whole-genome expression technologies. Therefore, we here integrated the physiological, metabolic and transcriptional analyses of DOKM seedings subjected to CA and control. Our goal was to reveal the transcriptomic and metabolic responses under CA in DOKM. Consequently, we constructed a gene to metabolite network on basis of the observation on massive transcriptome and metabolome reprogramming during CA, further uncovered a comprehensive gene regulatory mechanism that involves cold signal perception and transduction, transcriptional regulation and gene expression. Finally, we also identified a large number of potentially interesting genes which will help to enhance cold resistance of DOKM in chilling growth conditions.

## Materials and Methods

### Plant Materials and CA Treatments

To minimize the influence of genetic variation, a mature capsule of *D. officinale* was initially obtained from Yandang cultivated population (28.37°N, 121.03°E; Zhejiang Province, China). The seeds in capsule were subsequently sown into culture bottles (10-cm diameter) containing 120 mL of 1/2 MS medium (0.5 g L^–1^ NAA, 7 g L^–1^ agar and 30 g L^–1^ sucrose; pH 5.8-6.0) and further incubated in a culture room (12 h of daylight, 60 μmol m^–2^ s^–1^, 20 ± 1°C). Germinated seedlings were sub-cultured in the above medium every 45 days and five seedlings were planted in each culture bottle. After 120 days growth, seedlings with roots were further transferred to four identical controlled-climate chambers for both CA and control treatment. Within CA groups, the seedlings were placed in chambers set at 4, 0, and –2°C CA treatments (12 h/12 h light/dark, 80% humidity, with 60 μmol m^–2^ s^–1^ photosynthetically active radiation), respectively. The control seedlings were located in a 20°C chamber with other parallel growth conditions as CA treatment.

To obtain a full transcriptomic and metabolomic responses to CA, the physiological analyses were firstly performed for confirming a fitness CA point, and leaves were harvested from each time points (4, 12, 16, 20, and 24 h) in each temperature stage for CA and control. Consequently, leaves were collected at 0°C during 20 h and 20°C during 20 h (control) for transcriptome and metabolome analyses (see Results). Each sample consisted of leaves from five plants grown in the same bottle.

### Measurement of Antioxidant Enzyme Activities and Electrolyte Leakage (EL)

Fresh leaf samples (1.0 g) from CA treated and control plants were homogenized with an extraction buffer containing 100 mM potassium phosphate buffer (pH 7.8), 0.1% Triton X-100 and 1% polyvinylpyrrolidone (PVP) using pre-chilled mortar and pestle. The homogenate was centrifuged at 10,000 ×*g* for 20 min at 4°C. The supernatant was used for enzyme assays. The activities of antioxidant enzymes (superoxide dismutase, SOD; catalase, CAT; peroxidase, POD; ascorbate peroxidase, APX) were quantified, respectively, using assay kits (A001-1, A007-1, A084-3, A123; Nanjing Jiancheng, China) following the manufacturer’s instructions as described previously (Li et al., 2013). The activities were expressed as units (U) per g fresh weight (FW) of sample. EL was determined according to the plant CA methods and protocols as described previously (Thalhammer et al., 2014). In brief, leaf tissue samples (5-mm diameter) were washed in deionized water and placed in closed tubes with 10 mL of deionized water at 25°C for 2 h. After incubation, the conductivity in the bathing solution was determined (*L*_1_) using a conductively meter (Leici-DDSJ-318, Shanghai, China). Afterward, the samples were heated at 100°C for 25 min and the conductivity in the bathing solution (*L*_2_) was determined again. EL was calculated as follow: EL = *L*_1_/*L*_2_ × 100%. All physiological assays for each point were carried out three independent replicates. The statistical differences among different treatments were assessed by Tukey’s test at 99% of confidence level (*P* < 0.01) using SPSS software version 16.0 (SPSS Inc., USA).

### Metabolite Profiling Analysis

Hundred milligram of frozen leaf sample in liquid nitrogen was extracted in 4 mL methanol solvent with 150 μL internal standard [0.2% (m/v) ribitol in water]. Extraction was performed at 60°C for 20 min and the extractive was centrifuged at 14,000 *g* for 5 min. The liquid fraction was collected and 250 μL of chloroform were added. The mixture was further centrifuged at 5,000 *g* for 10 min and the supernatant was dried in a vacuum concentrator. Sample derivatization procedure was followed previously (Dethloff et al., 2014). The dried samples were derivatized at 30°C for 90 min with 40 μL of 2% (m/v) methoxyamine hydrochloride in pyridine. Next, metabolites were treated with 80 μL of *N*-methyl-*N*- (trimethylsilyl) trifluoroacetamide (Pierce, USA) dissolved in pyridine with a 7:1 (v/v) ratio. The derivatized extract was determined using an Agilent 7890A gas chromatograph with an Agilent 5975C inert XL EI/CI mass spectrometric detector (MSD) system (Agilent Technologies, Santa Clara, CA, USA). The internal standard (Ribitol) was used to normalize the metabolites over the samples.

Metabolite identification and annotation were performed by searching against commercial available databases such as National Institute of Standards and Technology (NIST05^1^) and Wiley libraries according to recommendations by Tohge and Fernie (2009) and Fernie et al. (2011). Ten independent biological replicates were performed for both CA and control treatments. Differentially accumulated metabolites were defined according to the following criteria: accumulation fold change > 1.5 and *P* < 0.01 based on Tukey’s test. Based on differentially accumulated metabolites, the hierarchical cluster analysis (HCA) was performed using the CLUSTER software package^2^ and Java Treeview^3^; principal component analysis (PCA) was also performed using SPSS software version 16.0 (SPSS Inc., USA) based on the transformed data of metabolite accumulation by means of zero-mean normalization.

### RNA Extraction, Library Construction, and Illumina Sequencing

The leaf samples from three independent biological replicates for both CA and control were harvested and frozen in liquid nitrogen. Total RNA of each sample was extracted using the RNAiso kit for polysaccharide-rich plant tissue (Takara, Dalian, China) and purified using DNase1 (TURBO DNase, Ambion, USA) to avoid DNA contamination. RNA Integrity Number (RIN) values (>8.5) were determined using a Bioanalyzer 2100 (Agilent Technologies, Santa Clara, CA, USA). The sequencing libraries were constructed using a cDNA Synthesis kit (Illumina Inc., San Diego, CA, USA) following the standard Illumina preparation protocol. Paired-end (2 × 125 bp) sequencing of the cDNA libraries was performed on the Illumina HiSeq 2500 (Illumina Inc., San Diego, CA, USA) by the Biomarker Biotechnology Corporation (Beijing, China). Libraries from each biological replicate yielded more than 6 GB of raw data. The raw reads of transcriptome and their transcript assemblies have been deposited into NCBI Sequence Read Archive (SRA) and Transcriptome Shotgun Assembly Sequence database (Accession: GEZV00000000) under the BioProject number PRJNA 314400.

### *De novo* Transcriptome Assembly and Functional Annotation of the Transcriptome

Raw reads were firstly preprocessed by removing those reads with only adaptor and unknown nucleotides >10%, and low-quality reads with quality scores less than Q30. Next, *de novo* assembly was performed using the high-quality reads obtained in the Trinity platform (released^4^ 2011-05-19) with the parameters “*K*-mer = 25, group_pairs_distance = 500, min_glue = 2, min_kmer_cov = 2” (Grabherr et al., 2011). Subsequently, the TGICL software system was used to cluster the potential unigene and form a single set of non-redundant unigene (Pertea et al., 2003).

Annotation of all assembled unigenes was carried out by matching against public databases [Nr, Swiss-Prot, Gene Ontology (GO), COG, KOG, Kyoto Encyclopedia of Genes and Genomes (KEGG)] using the BLASTX analysis with a cut-off *E*-value of 10^–5^. Based on the BLASTX output (xml format), the Blast2GO software (version 2.3.5) was further used to assign GO terms by describing biological processes, molecular functions, and cellular components (Götz et al., 2008). To identify which GO terms are enriched within all CORs, the R package topGO was employed to evaluate the significance of enrichment of each GO term using the default algorithm and Kolmogorov–Smirnov (KS) test (*P*-value ≤ 0.01) (Alexa et al., 2006). Furthermore, the unigene sequences were compared with the COG database to predict and classify possible functions. For differentially expressed genes (DEGs), Kyoto Encyclopedia of Genes and Genomes (KEGG) pathway enrichment analysis was conducted using KOBAS 2.0^5^ with a Bonferroni-adjusted *P*-value ≤ 0.01 (Xie et al., 2011). In addition, transcription factors (TFs) were classified according to the *Arabidopsis* TF database (Perez-Rodriguez et al., 2010).

### Global Expression Analysis of Transcriptome

For gene expression analysis, all usable reads were mapped with the assembled transcriptome using TopHat (version 2.0.8), and then normalized into FPKM (Fragments per kilobase of exon per million fragments mapped) values using cuﬄinks (version 2.1.1) (released 2013-04-11^6^) (Mortazavi et al., 2008). DEGs were further characterized and estimated using the R software module edgeR (R v2.14; edgeRv 2.3.52) according to the results from cuﬄinks (Robinson et al., 2010). False discovery rate (FDR) <0.01 and an estimated absolute log_2_ fold-change (log_2_ FC) ≥ 1 were used as threshold for determining significant difference in gene expression between CA and control. To depict a comprehensive gene to metabolite network associated with CA dynamic changes, all DEGs generated were assigned to metabolic pathways using the KEGG^7^ annotation. Additionally, the Pearson correlation coefficient between three independent replicates from an identical sample processing method was calculated with R software (version 3.1.3) for verifying the gene expression profiles. HCA of DEGs was also performed according to Eisen et al. (1998) based on the FPKM value (log_2_ -transformed data) for each gene.

### Verification of Quantitative Real-Time PCR (qRT-PCR)

Genes of interest were selected for further confirmation by qRT-PCR analysis following the manufacturer’s protocols (SYBR Green I; Lumiprobe). Total RNA was extracted and purified as above. Gene specific qRT-PCR primers were designed using Primer-BLAST (http://blast.ncbi.nlm.nih.gov/Blast.cgi), and the sequences are listed in Supplementary Table S4 in Supplementary Material. The qPCR reactions were performed as described in Guo et al. (2013). All quantifications were normalized to the EF-1a gene (Accession: JF825419) and calculated using the 2^–ΔΔ^Ct method (Livak and Schmittgen, 2001). Three biological replicates were used for qRT-PCR analysis.

### Gene Network Construction and Visualization

To gain more insight into the interaction of CORs during CA, a gene interaction network was built using Cytoscape software (v2.8.3) (Smoot et al., 2011). The potential interaction data for all cold regulated genes (2,767 genes; see results) were extracted from the latest STRING database (STRING v10) (Szklarczyk et al., 2015). The interactions with a confidence score ≥400 between two genes were kept and imported into Cytoscape for visualization. The Cytoscape plugin MINE was used to identify the major interaction hubs where pathways converge (Rhrissorrakrai and Gunsalus, 2011).

## Results

### Cold-Induced Changes in Antioxidant Enzyme Activities and Electrolyte Leakage during CA

**FIGURE 1:**
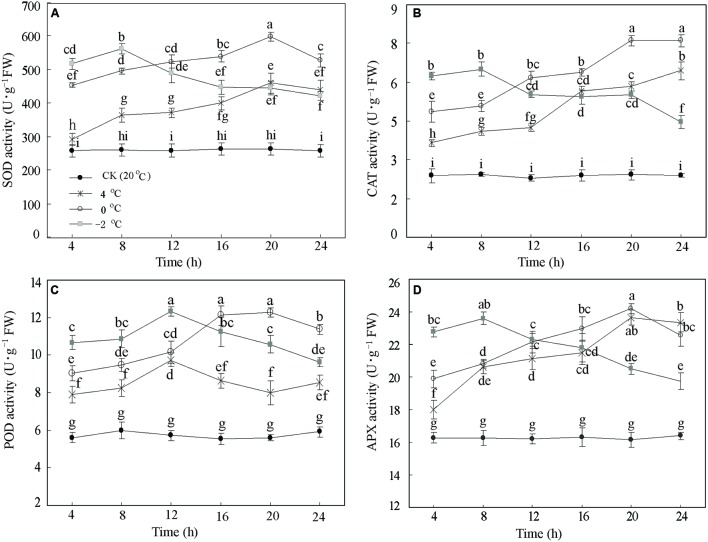
**Antioxidant enzymes responses of *Dendrobium officinale* to cold acclimation.** The activities of **(A)** superoxide dismutase (SOD) activity, **(B)** catalase (CAT) activity, **(C)** peroxidase (POD) activity, and **(D)** ascorbate peroxidase (APX) were quantified by the means of three independent experiments ± SD. Different letters mark indicates significantly different at *P* < 0.01.

To assess whether low temperature alters activities of several antioxidant enzymes in DOKM seedlings, we monitored the responses to CA at 20°C (Control), 4, 0, and –2°C during 24 h. Lowering temperature significantly (*P* < 0.01) increased the enzyme activities of SOD, CAT, POD, and APX. The enzyme activities showed a rise-and-drop tendency as time progressed (**Figure 1**). For example, the SOD activities at 4 and 0°C displayed an almost linear increase until 20 h (459.33 and 597.94 U g^–1^ FW, respectively), and thereafter decreased to 439.18 and 527.95 U g^–1^ FW, respectively. Further, reduction of temperature to –2°C significantly enhanced the SOD activity compared to 4 and 0°C during the first 8 h. However, the activity sharply decreased after 8 h and ended up at 422.08 U g^–1^ FW at 24 h (**Figure 1A**). Similar patterns were also drawn from changes of CAT, POD, and APX (**Figures 1B–D**). Amongst all time points of CA, it is interesting to note that all activities of the four enzymes at 0°C during 20 h were the highest as compared with the control.

**FIGURE 2:**
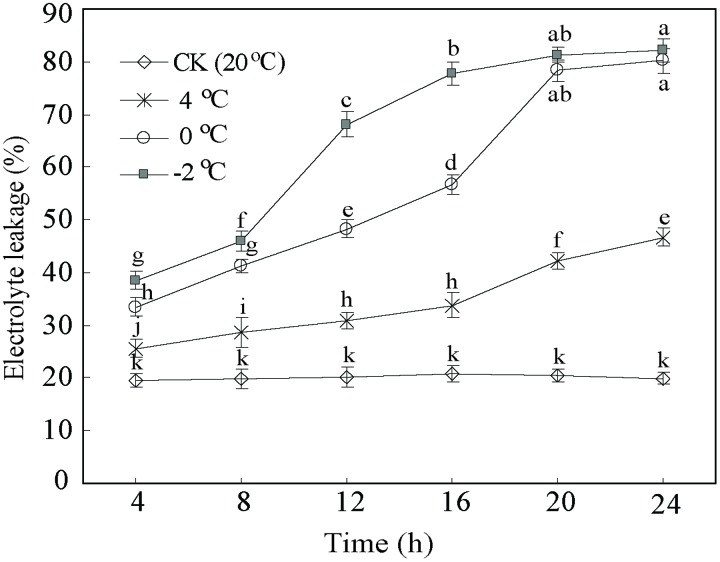
**Cold tolerance expressed as electrolyte leakage of *D. officinale* under cold acclimation and control.** Results are shown as means ± SD from three independent experiments. Different letters mark indicates significantly different at *P* < 0.01.

In addition, EL as a suitable tool allows assessing the level of cold tolerance. **Figure 2** illustrates the expected differences in the EL curves between CA treatments and control. The EL from control samples were stable and lower (*c*. 20%) than CA treatments. By contrast, the EL of the CA samples at 4°C displayed an increase from 25.5 to 48.4%. Particularly, the EL at 0 and –2°C notably increased from 33.6 to 80.1%, and from 39.1 to 80.9%, respectively. Interestingly, EL showed a two fold increase until 20 h at 0 and –2°C, subsequently kept stabilized. This suggested that this stage is a saturate point for maintaining the membrane integrity during cold stress. Considering the whole data set at the four different temperatures and five time-points, we decided to take 0°C during 20 h as the optimum CA treatment and 20°C during 20 h as control for cold stress. The two experimental points had the biggest differences in physiological parameters (**Figures 1** and **2**), which enables obtaining a full transcriptomic and metabolomic responses to CA.

### Metabolome Reprogramming under CA As Compared with Control

**FIGURE 3:**
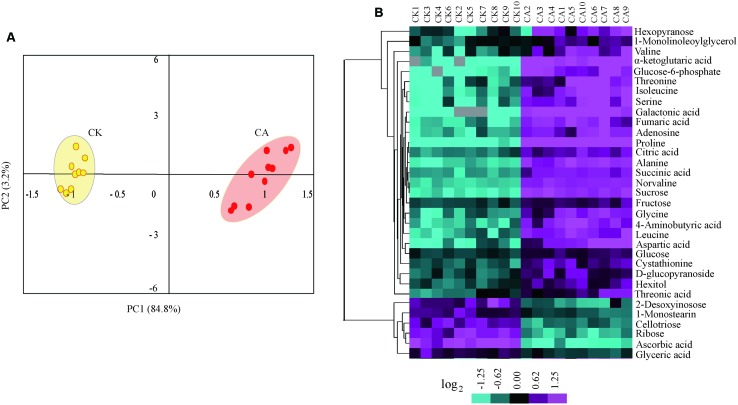
**Metabolome reprogramming during cold acclimation (CA) in comparison with control.** Shown are the metabolome profiles for ten independent biological replicates of CA and parallel control replicates (CK). **(A)** Principal component analysis (PCA) depicted a metabolome reprogramming during CA as compared with control. Values for *x* and *y* axes are scores for PC1 and PC2. **(B)** Hierarchical cluster analysis (HCA) of all differentially accumulated metabolites between CA and control treatments. The bar at bottom represents the color code for log_2_-transformed data of metabolite accumulation using the CLUSTER software package. The levels of metabolite accumulation are listed in Supplementary Table S1 in Supplementary Material.

To enable capturing a comprehensive picture of metabolite reprogramming during CA, we performed metabolite profiling of ten parallel samples from CA treatment (at 0°C during 20 h) and control using GC-MS. We reproducibly detected a total of 68 metabolites in leaf samples. Of these, 33 metabolites showed differential accumulation (Fold change > 1.5, *P* < 0.01), namely 27 increased and 6 decreased under CA compared with control (Supplementary Table S1 in Supplementary Material). Based on the differentially accumulated metabolites, we performed PCA and a HCA heat map to investigate the temporal accumulation patterns of these metabolites in responses to CA. The first two principal components clearly separated 20 samples from CA and control and explained 88.0% of the total variation in entire data set. The dimensional separation indicated distinct differences in the levels of metabolites (**Figure 3A**). Specially, PC1 (84.8%) predominantly reflected cold-triggered differences in metabolites including amino acids, organic acids and sugars. The majority of these metabolites accumulated significantly higher levels during CA in comparison with control, including 9.9-fold proline, 4.3-fold alanine, 4.2-fold isoleucine, 4.1-fold serine, 4.1-fold aspartic acid, 21.6-fold a-ketoglutaric acid, 13.7-fold galactonic acid, 3.9-fold fumaric acid, and 4.6-fold sucrose (Supplementary Table S1 in Supplementary Material). Furthermore, the metabolite profiles were reprogrammed and reflected by PC2 (3.2%), which was characterized by high level of monostearin and low level of deoxyinosose. Similarly, HCA (**Figure 3B**) also presented the strong responses to CA on metabolite levels, and distributed metabolites into two major clusters representing similar accumulation profiles. This massive metabolic adjustment reflected the full activation of CA-related responses.

Interestingly, these differentially accumulated metabolites represented distinct metabolic categories based on their KEGG classification. In this study, 10 amino acids, four sugars and seven organic acids were annotated into the carbon metabolic pathway involving glycolysis, tricarboxylic acid (TCA) cycle and amino acid metabolism. This indicated a direct link between these pathways and the response to CA. Overall, these data clearly demonstrated that the metabolic reprogramming is a tightly regulated process during CA.

### Acquisition of Transcriptome Data by *De novo* Assembly

To enable transcriptomic comparison of the acclimation response to cold with control, we characterized the transcriptome of DOKM under CA and control conditions using RNA-seq. **Table 1** presents the overview of RNA-Seq reads from six libraries; a total of 177,183,150 clean reads were obtained from all libraries. The probability of incorrect base calling was used to evaluate the sequencing quality by the value of ≥Q30, the high proportion of ≥Q30 (∼86%) for each library showed a high quality of RNA-seq. Finally, these high-quality reads were *de novo* assembled into 150,349 contigs and further generated 79,256 unigenes using Trinity program (Grabherr et al., 2011). The assembly generated a large amount of smaller sized unigenes, the details of size distribution of these contigs and unigenes are shown in Supplementary Figure S1 in Supplementary Material. Furthermore, we mapped our RNA-Seq reads to the assembled transcripts to estimate the efficiency of short-read usage during *de novo* assembling. Over 81% sequences in each library were matched (**Table 1**), indicating that the set of assembled transcripts was appropriate for differential expression analysis.

**Table 1:** Numbers of RNA-seq reads mapping back to the *Dendrobium officinale* transcriptome.

Replicate	Number base in dataset (after QC)	Number clean reads	Number mapped to transcripts	% clean reads mapped to transcripts	% ≥ Q30
CK1	7,588,821,514	30,119,955	24,714,486	82.05	86.98
CK2	7,312,310,317	29,024,080	23,757,479	81.85	87.51
CK3	7,417,433,123	29,440,686	24,216,038	82.25	87.24
CA1	7,803,250,608	30,974,131	25,506,375	82.35	87.89
CA2	6,999,788,856	27,783,980	22,944,294	82.58	87.76
CA3	7,517,144,576	29,840,318	24,708,355	82.80	87.68
Total	44,638,748,994	177,183,150	145,847,027	82.31	–

*CK refers to the group of control; CA refers to the group of cold acclimation.*

Since DOKM is a non-model plant species, to acquire the most informative and complete annotation, we matched all assembled unigenes against public databases by BLASTX (*e* ≤ 1.00 × 10^–5^). Out of the 79,256 unigenes, 29,013 generated significant BLAXTX hit to genes encoding proteins or putative function in at least one of public databases (Supplementary Table S2 in Supplementary Material). In comparison with previous publication for DOKM (Guo et al., 2013), the low rate of annotated genes indicated that assembled unigenes, particularly sequences without a significant homologous hit, may potentially represent special sequences expressed during CA.

### CA-Associated Global Changes in Gene Expression in DOKM Seedlings

The gene expression profile was analyzed using uniquely mapped reads to estimate normalized transcription level as FPKM. Meanwhile, the expression values were ascertained using three independent biological replicates for both treatments (Supplementary Figure S2 in Supplementary Material). The expression values between the same treatments were highly correlated (*r*^2^ > 0.83), indicating that all collected samples were well processed. Next, a fold change ≥ 2 and false discovery rate (FDR) <0.01 were used as cutoffs to identify differentially regulated genes. In total, 1,331 genes were up-regulated and 1,436 were down-regulated during CA as compared with control.

**FIGURE 4:**
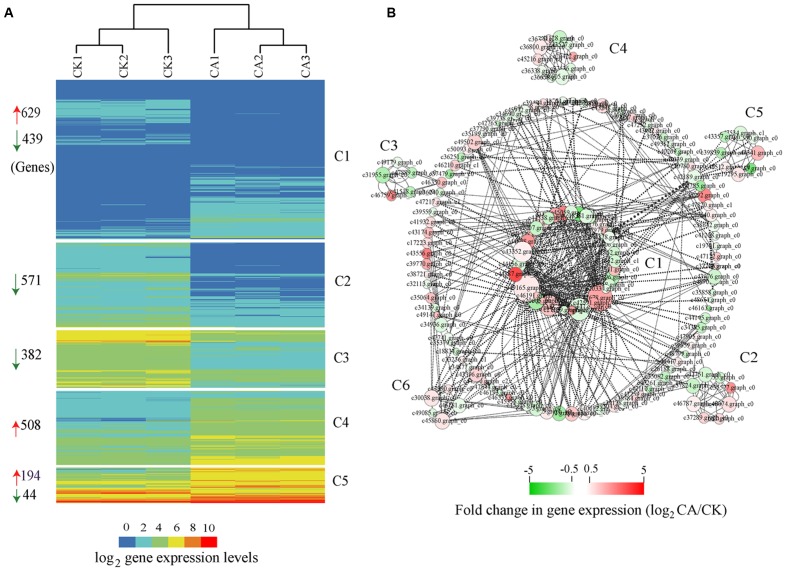
**Global expression profiles and interaction network of differentially expressed genes (DEGs) during cold acclimation (CA) as compared with control growth. (A)** HCA, indicating that all DEGs were grouped into five clusters (C1–C5) based on their expression levels. The numbers at the left of the map represent the amount of DEGs in each cluster. The red and green arrows mean up-regulation and down-regulation. **(B)** Interaction networks depicted multiple hubs (C1–C6) of gene interaction under CA condition. The network was generated based on the potential interaction data for 2,767 DEGs, which were extracted from the latest STRING database (STRING v10). Node size indicates its connectivity measured as node degree (i.e., the number of edges connecting the node); the bigger node means a higher connectivity. The edge (the connecting line) indicates the interaction between two genes, and the weight of edges between them is a measure of their interaction. Thick edges show strong interaction, whereas thin edges show weak interaction. The bar at bottom shows the color code for the fold change in gene expression of log_2_ -transformed data. All interaction data are available in Supplementary table S5 in Supplementary Material.

Based on HCA, we clustered all cold-induced genes into 5 co-expression clusters, each of which contained genes with similar expression pattern (**Figure 4A**). The expression profiles of three independent biological replicates for each treatment are showed. Among 2,767 cold-induced genes, we preferentially noted genes with higher expression levels (FPKM value > 10) and distinct differences (FC > 2) between CA and control. These genes, best represented by C4 and C5, were associated with transcriptional regulation, signal transduction, posttranslational modification, cell wall biogenesis and organization, energy production and conversion, carbohydrate transport and metabolism, and stress–related genes. In particular, some of the major marker genes exhibited significant up-regulation during CA, such as genes encoding DREBs, LEA, heptahelical transmembrane proteins, RING-H2 and zinc finger proteins, heat shock proteins (HSPs), protein kinases, glycosyl hydrolase family proteins, ATPases, sugar and phosphate transporters (Supplementary Table S3 in Supplementary Material). In addition to a large set of up-regulated genes, a large number of down-regulated genes were observed and represented by C1, C2, and C3. These genes were associated with plant response, protein ubiquitination, lipid transport and metabolism, including typical genes encoding defensin-related proteins, F-box/kelch motifs, phospholipase, lipoxygenase, and cytochrome P450 (Supplementary Table S3 in Supplementary Material). Overall, these results demonstrated that the major biochemical processes during CA were influenced partly by highly coordinated transcript reprogramming.

Furthermore, qRT-PCR was performed to provide a more rigorous quantitative measure of gene expression. Here, 20 marker genes encompassing different CA responses from initial signal sensing to target proteins, were selected and analyzed by qRT-PCR (Supplementary Table S4; Supplementary Figure S3 in Supplementary Material). These genes, transcriptional regulators such as CBF, ICE, HSP, protein kinases [mitogen-activated protein kinase (MAPK), CBL, EFR], LEA protein, showed very striking expression increase during CA in comparison with control. Moreover, a distractingly good agreement between RNA-seq and qRT-PCR analysis was found with a high linear correlation (*R*^2^ = 0.86; Supplementary Figure S3 in Supplementary Material), confirming the reproducibility of the gene expression profiles.

### Interaction Network Analysis of Cold-Induced Genes

An alternative analysis tool, gene interaction network, was adopted to understand the functional relationships and identify the major hubs between DEGs. Here, we constructed an interaction network for 2,767 DEGs on basis of known and predicted protein–protein interactions from the new STRING 10 available database. All genes harboring potential interaction relationships (Source-Target) are shown in Supplementary Table S5 in Supplementary Material. By using Cytoscape software (Smoot et al., 2011) and the plugin MINE (Rhrissorrakrai and Gunsalus, 2011), we loaded 168 nodes (genes) and 488 edges (interactions) and identified six clusters of strongly associated genes within the interaction network (**Figure 4B**).

Hub genes are those that show most connections in the network. The network, in combination with the GO function analysis and KOG class annotation, revealed a strong association between genes involved in cold stress signal transduction by encoding multiple receptor kinases, cellular response to stress stimulus by protein folding, and other genes involved in protein phosphorylation, transcription. Many genes implicated in CA were predominantly presented in cluster 1 (C1) of **Figure 4B**, such as 2C-type protein phosphatases (PP2C5, Os02g0224100, and At2g30020), MAPK signal transduction cascades (MPK5, MPK20, and MAPKKK16), CDPK gene families related with Ser/Thr protein kinases (STN7, WAG1, and RPK2), heat shock proteins (Hsp70, HSP90.7) and DNAJ protein (ERDJ3A), and regulators related with C2H2-type zinc fingers (ZAT10, ZAT11, and MAGPIE), MADS-box region (MADS16, SEP1) and bZIP TF (HY5). Furthermore, genes involved in calcium and calmodulin signaling network were the core components of C2, C3, most of which are represented by CDPK gene families (IRE4, bam1, ERECTA, HSL1, At1g74360, and IKU2) and MAPK cascade (MKK9) (Supplementary Table S6 in Supplementary Material). Genes involved in chloroplast development play a critical role in adapting stress environments (Yin et al., 2012). Interestingly, the C4 cluster contained multiple marker genes associated with protein synthesis by maintaining the chloroplast membrane system, including chloroplast ribosome proteins (RPL3, RPL5, RPL23, RPL28C, and RPL4A), thylakoidal processing peptidase 1 (TPP1), elongation factor TU GTP-binding domain (Eftud1). Additionally, cluster 5 contained many known genes involved in plant hormone biosynthesis and degradation (encoding cytochrome P450 family proteins), and lipid metabolism, including typical gene CYP707A1, CYP72A8, CYP90B1, and CYP85A2.

### Functional Categories of Genes Affected by CA in DOKM Seedlings

To further understand the functional significance of genes and their products associated with CA, we annotated molecular function for all DEGs by using GO database. Among 1,150 genes with at least one GO terms assigned, 823, 853, and 664 genes were annotated into the biological process, molecular function, and cellular component categories, respectively (Supplementary Figure S4 in Supplementary Material). In light of GO enrichment analysis, we found that the most strikingly GO terms enriched (*P* < 0.01) were related to cellular protein modification process, phosphorylation, carbohydrate derivative biosynthetic process; ATP binding, protein kinase activity, protein serine/threonine kinase activity, catalytic activity; cytoplasmic membrane-bounded vesicle, integral to plasma membrane (Supplementary Table S7 in Supplementary Material).

Next, we matched all DEGs against COG database to predict and classify its possible functions. Supplementary Figure S5 in Supplementary Material shows the distribution of COG functional categories amongst up-regulated genes by CA. The most prominent functional groups were general function prediction only, signal transduction mechanisms, transcription, carbohydrate transport and metabolism, amino acid transport and metabolism. Additionally, the KEGG pathway classification allowed giving deeper insights into the biological process affected by CA. By using all DEGs as objects to search against KEEG pathway maps in *Arabidopsis thaliana*, 225 genes were annotated and assigned to 86 metabolic pathways. Interestingly, in the responses to CA, several significantly enriched (*P* < 0.01) pathways for DEGs were linked to arginine and proline metabolism (Ko00330), alanine, aspartate and glutamate metabolism (Ko00250), citrate cycle (ko00020), starch and sucrose metabolism (Ko00500), plant hormone signal transduction (Ko04075) (Supplementary Table S8 in Supplementary Material).

### Characterization of Genes Encoding Transcription Factors (TFs)

Based on the *Arabidopsis* Information Resource (TAIR), we surveyed the biological functions of putative TFs that were differentially expressed between CA and control. Of all 81 families of TFs predicted from the *Arabidopsis* TF database (Perez-Rodriguez et al., 2010), we detected 52 families with at least one gene matched to DEGs dataset. In total, 1,018 genes as TFs were differentially expressed, with 481 up-regulation and 537 down-regulation (Supplementary Table S9; Supplementary Figure S6 in Supplementary Material). Interestingly, this accounted for 37.0% of the total DEGs, implying their important roles in regulating genes involved in cold responses.

In particular, families showing especially strong responses to CA included bHLH, AP2-EREBP, HD-ZIP, bZIP, NAC, B3, WRKY, MYB, C2H2, HSF, and C3H with over 30 family genes. During CA, special regulation was noticed for members of AP2-EREBP, HD-ZIP, bZIP, HSF, C2H2, and bHLH families. For instance, some genes from these families were more than 20-fold up-regulation during CA as compared with control. By contrast, some TFs belonging to B3, GEBP, and TCP families showed remarkable down-regulation. This demonstrated that these TFs as preferentially expressed genes under CA governed the massive and highly coordinated transcriptional changes through both, transcriptional activation and repression.

## Discussion

### The Energy Homeostasis Program during CA Is Strongly Associated with Carbohydrate and Amino Acid Metabolic Responses

The requirement to generate energy is an intrinsic feature of cold stress responses (Huner et al., 1998). In plants subjected to CA, to enhance adaptability of plant to low temperature, high levels of sugars and ATP (and other energy carriers) must be timely used to synthase COR proteins, lipids and membrance compositons, and to further promote cell membrane transport and structural rearrangement (Dobrota, 2006; Fristedt et al., 2015). In this study, such marker reflecting ATP requirement in the energy homeostasis system was the up-regulation of a variety of energy carrier proteins such as 91.4-fold ATPUMP5 (c19814.graph_c0), 9-fold ATP/GTP-binding protein (emb1579, c38770.graph_c0), and 32.2-fold ATBT1 (c47416.graph_c0) during CA (Supplementary Table S3 in Supplementary Material), which were involved in energy transfer. As anonther important envet of maintaining energy balance, adenylate kianase (ADK, c49278.graph_c1) plays a crucial role in regulating the right equilibrium between ADP and ATP levels (Lange et al., 2008), which presented 2.6-fold up-regulation in CA treated seedlings. Moreover, ATP can be an important signaling molecule and generate the proton-motive force to push the Ca^2+^ influx across the plasma membrane (Kim et al., 2006). Here, this action was strongly supported by 3.8-fold overexpression of an typical calcium-transporting ATPase (ECA2c, 49194.graph_c0) during CA.

**FIGURE 5:**
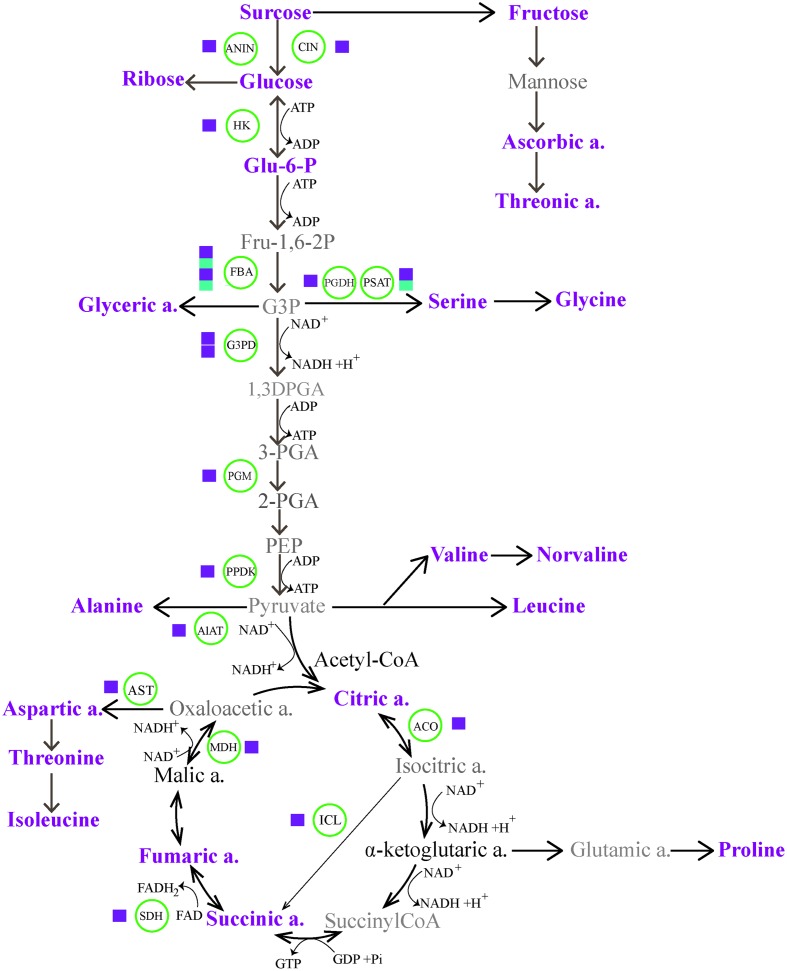
**The energy homeostasis strategies through carbohydrate and amino acid metabolic pathways, indicating massive transcriptome and metabolome reprogramming during cold acclimation (CA).** The magenta and cyan squares represent up-regulation and down-regulation of a biosynthetic gene. The metabolites in magenta represent up-regulated accumulation under CA compared with control. The metabolites in bold show no differences in accumulation, and the metabolites in gray are not identified by GC-MS. ACO, aconitate hydratase; AlAT, alanine aminotransferase; ANIN, alkaline and neutral invertase; AST, aspartate aminotransferase; CIN, cell wall invertase; FBA, fructose-bisphosphate aldolase; Glu-6-P, glucose-6-phosphate; G3DP, glyceraldehyde 3-phosphate dehydrogenase; G3P, glyceraldehyde-3-phosphate; HK, hexokinase; ICL, isocitrate lyase; MDH, malate dehydrogenase; PEP, phosphoenol pyruvate; 3-PGA, 3-phosphoglycerate; PGDH, D-3-phosphoglycerate dehydrogenase; PGM, phosphoglycerate mutase; PPDK, pyruvate phosphate dikinase; PSAT, phosphoserine aminotransferase; SDH, succinate dehydrogenase.

In nature, plants commonely exploit any one or a combination of mulitple mechanisms (such as photosynthesis, producing pyrophosphate, adjusting metabolic sinks, etc.) to offset the potential energy cirisis during CA (Huner et al., 1998; Dobrota, 2006). Of particular interest in the context of energy supplies is switching energy metabolism. Here, to achieve a more comprehensive understanding of CA dynamic event, we combined transcriptomic and metabolic data for constructing a gene to metabolite network. As shown in **Figure 5**, the associated massive transcriptome and metabolome reprogramming that we observed during CA activated multiple alternative proceses to generate energy. The first process involved increased glycolysis or oxidation of carbohydartes in central carbohydrate metabolism. In the process, Glc is a precursor in glycolysis and the glycolysis of Glc can generate ATP and NADH with pyruvate as the end product. The dramatical accumulation of Glc from Suc hydrolysis was highly facilitated by the genes encoding cell wall related invertase (CIN, c40269.graph_c0; ANIN, c48382.graph_c0), which presented more than four fold up-regulation during CA (**Figure 3**; Supplementary Table S1 in Supplementary Material). The up-regulated genes ensured a continuous supply of Glc for the energy cycle. Consistent with this result, the importance of CIN and ANIN in yielding Glc has been implicated in cold treated *Arabidopsis* and rice plants (Oliver et al., 2007; Maruyama et al., 2014). In addition, the strong transcriptional activation of other genes encoding enzymes involved in the next steps of glycolysis were also observed. These genes contained hexokinase (HK), fructose-bisphosphate aldolase (FBA), glyceraldehyde 3-phosphate dehydrogenase (G3P); phosphoglycerate mutase (PGM), and pyruvate phosphate dikinas (PPDK). Remarkably, they showed higher expression levels in CA treated seedlings than contol, suggesting they can facilitate bypassing the ATP-generating steps of glycolysis during CA (Oufir et al., 2008; Jozefczuk et al., 2010).

Alternative form of a switch to energy metabolism is associated with amino acids biosynthesis and TCA cycle. In the process, pyruvate can be degraded into branced amino acids (such as Ile, Leu, and Val) and be further metabolized in TCA to produce acetyl-CoA, which is an essential precursor in the synthesis of secondary metabolites. The catabolism of Ile, leu, Val and progressive conversion of organic acids in TCA with intial help of acetyl-CoA results in the production of high energy carriers (such as NADH, FADH2, and GTP). Such a process has been widely observed during CA and other stress conditions (Kaplan et al., 2007; Jozefczuk et al., 2010; Shi et al., 2013). Our results showed that the accumulation of several important intermediaries such as citratic acid, succinic acid and fumaric acid in TCA were responsible for the activation of multiple enzymes encoding isocitrate lyase (ICL), aconitate hydratase (ACO) and succinate dehydrogenase (SDH), which were more than two fold up-regulation during CA, especially ICL that was six fold up-regulated.

Notably, the metabolic reprogramme of carbohydrates and amino acids is not noly crucial to generate energy, but also these metabolites play direct roles in working as osmolytes or compatible solutes in the response to CA. The accumulation of these metabolites during CA contained multiple sugars (Suc, Glc, and Fru) and various amino acids such as Pro, Asp, Thr, ect. (Supplementary Table S1 in Supplementary Material), which are exemplified by a common occurrence in plants in response to abiotic stress and act as bulk osmoprotectants or ROS scavengers to stabilize membranes (Nishizawa et al., 2008; Keunen et al., 2013). In particular, a well studied metabolite is proline that can protect enzymes and other proteins from denaturation during CA (Savouré and Szabados, 2010). In this study, 10-fold up-regulation of proline in CA treated seedlings was helpful to enhance the flexibility of cold tolerance during CA. Taken together, the changes observed on transcriptomic and metabolic levels, the specifical up-regulation of metabolites and catabolic/biosynthetic enzymes from glycolysis, amino acid metabolism and TCA cycle not only directly provided various reagents to protect against cold demage, but also contributed to energy homeostasis during CA.

### Signaling Transduction and Regulation during CA

**FIGURE 6:**
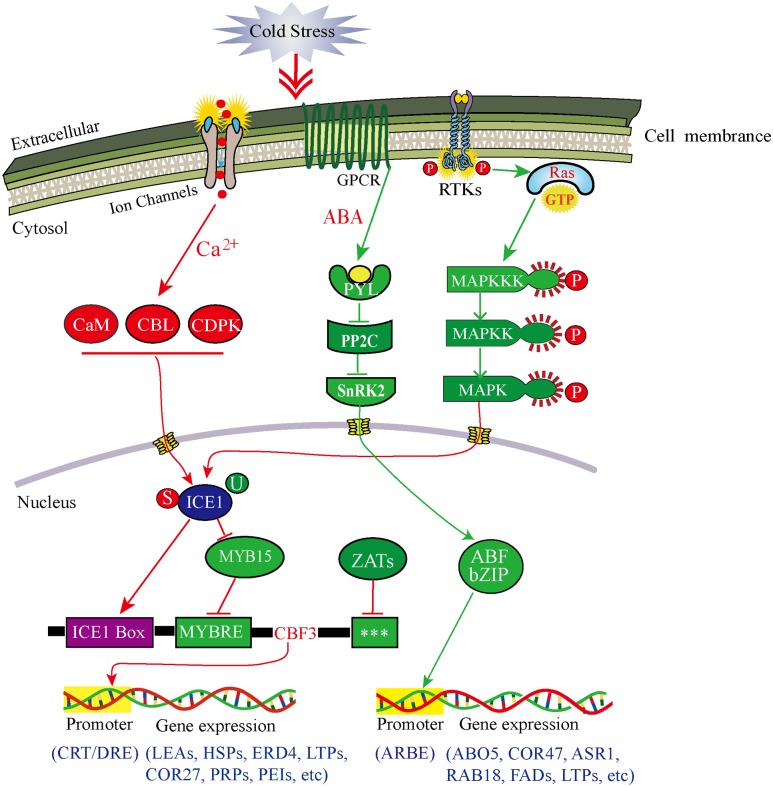
**Model of multiple signal transduction pathways and transcriptional networks during cold acclimation in *D. officinale*.** Red lines represent Ca^2+^-dependent signaling process and CBF-dependent regulatory pathway; green lines represent Ca^2+^-independent signaling process and ABA-dependent regulator pathway. Arrows represent positive regulation, whereas lines ending with a bar represent negative regulation. The three stars (^∗∗∗^) indicate unknown *cis*-elements. ABF, ABA-responsive element binding factor; ABO5, ABA overly- ensitive 5; ABRE, ABF recognition element; ASR1, ABA stress-ripening protein 1; CaM, calmodulin; CBL, calcineurin B-like Ca^2+^ sensors; CDPK, Ca^2+^-dependent protein kinase; COR27/47, cold regulated gene 27/47; CRT, C-repeat elements; DRE, dehydration-responsive elements; ERD4, dehydration-induced protein 4; FADs, fatty acid desaturases; GPCR, G-protein-coupled receptor; HSPs, heat shock proteins; LEAs, late embryogenesis abundant protein; LTPs, lipid-transfer proteins; MYBRS, MYB transcription factor recognition sequence; PEIs, pectinesterase inhibitors; PP2C, 2C-type protein phosphatase; PRPs, proline-rich proteins; PYL, pyrabactin resistance 1-like; RAB18, dehydrin family protein 18; RAS, rat sarcoma protein; RTKs, receptor-tyrosine kinases; SnRK2, SNF1-related protein kinase 2; P, phosphorylation; S, sumoylation; U, ubiquitination.

Our transcriptomic data, combined with the interaction network analysis of DEGs, clearly showed that many of genes containing receptors, phosphorylation kinases and TFs, were involved in singal perception and tranduction in the response to CA (Supplementary Table S6 in Supplementary Material). To further demonstrate how DOKM seedlings connect cold signal recognition and tranduction with transcriptional regulation to optimize survival during CA, we construced a comprehensive model for signaling leading to cold-responsive transcriptional network based on our transcriptomic data (**Figure 6**). Several lines of evidence have established that an immediate increase in cytosolic calcium through membrane rigidification or ligand-activated Ca^2+^ channel, is one of the major signaling events triggered by cold stress (Carpaneto et al., 2007; Zhu et al., 2013). Here, we identified a large number of up-regulated genes econding calmodulins, CBLs and CDPK family proteins during CA, such as 22-fold CML 38 (c36307.graph_c0), 19-fold CML19 (c43556.graph_c0), and multiple more than 4-fold Ser/Thr protein kinases (c49141.graph_c0, c46935.graph_c0, c45676.graph_c0) as kinase domain of CDPK (Supplementary Table S3 in Supplementary Material). These genes are ideal candidates for perceiving intracellular changes in Ca^2+^ level and translating them into specific phosphorylation events to initiate downstream signaling processes (Reddy et al., 2011; Schulz et al., 2013).

Additionally, mitogen-activated protein kinase (MAPK) cascade organized by three-tiered modules (MPKKK-MPKK-MPK) is considered to be an evolutionarily conserved module involved in cold signal transduction (Cristina et al., 2010). Here, the outstanding finding is that one initial genes econding MAPKKK16 (c44887.graph_c0) presented a remarkable up-regulation (56-fold) during CA. Consequently, the MAPK pathway could be in turn activated by MAPKKK16 phosphorylation (**Figure 6**). Such change was associated with the activation of both, a small GTPases (Ras-related protein, c44117.graph_c0) and multiple up-regulated RLKs (especially receptor tyrosine kinase) through connecting the MAPK module to external cold stimuli (Nan et al., 2015).

On the other hand, the phytohormone abscisic acid (ABA) plays a key role in regulating plant response to both biotic and abiotic stress through a double negative regulatory system (PYR/PYL-| PP2C-| SnRK2) (Ma et al., 2009; Park et al., 2009). Here, two up-regulated PYL4 genes (c13547.graph_c0, c35332.graph_c0) and one down-regulated PYL2 gene (c33173.graph_c0) as ABA-receptors were identified and involved in the ABA-induced signaling pathway (**Figure 6**). These genes can negatively regulate the expression of 2C-type protein phosphatases (PP2Cs), which further allow SnRK2s to keep an inactive state by phosphorylation (Ng et al., 2011; Soon et al., 2012). Of particular interest was the seven special genes encoding PP2Cs such as ATPP2CA, PP2C_12/24/32, which were more than 2.5-fold down-regulated during CA. Consistent with what was reported during other plant cold stress (Antoni et al., 2012; Bhyan et al., 2012), this inhibition can enable SnRK2s to gain full activity by activation loop auto phosphorylation (Soon et al., 2012). Subsequently, two genes were predicted to encode SnRK2s (SNRK2-3; SNRK2-10) with more than five fold up-regulation during CA, which allowed intensifying activation of the ABA-induced signaling pathway. Overall, the cold-induced response in this study suggested a mechanism, whereby plants can open multiple pathways to transduce cold signal to nucleus and then regulate gene expression.

### Major Regulators and Regulatory Mechanisms during CA

Our transcriptomic data depicted that the CA is an active process allowing DOKM to maintain the cellular homeostasis by regulating the transcriptional, post-transcriptional, translational and post-translational processes (**Figure 6**). Of particular interest were master regulators that govern the massive and highly coordinated transcriptional changes under CA. In line with other plant findings during CA, some of the major transcriptional regulators associated with the regulation of the overall transcriptome and metabolome of DOKM were CBF/DREB, AP2/EREBP, bZIP, MYBs, and multiple-type zinc finger proteins (Supplementary Figure S6; Supplementary Table S9 in Supplementary Material), indicating the complexity of the regulatory pathways during CA (Rasmussen et al., 2013; Li et al., 2015). Despite the presence of all these, and other TFs in different regulation hub, CBF/DREB from AP2/ERF super-family in particular play a critical role in regulating the cold-responsive transcriptome changes (Thomashow, 2010; Kurepin et al., 2013). This observation was strengthened by the fact that all four predicted CBF TFs presented dramatic activation (especially 153-fold up-regulation of c34296.graph_c0), which played a direct role in activating expression of downstream COR genes.

In upstream of the CBF regulatory hub, a widely known regulator is ICE1 that encodes a MYC-type basic helix-loop-helix (bHLH) TF, which directly binds to MYC *cis*-element in the promoter of CBF/DREB that interact with CRT/DRE of downstream of COR genes and regulate cold-induced responses (Chinnusamy et al., 2003). In this work, an ICE1 gene (c31676.graph_c0) was up-regulated, suggesting that CBF acted as a target of cold-induced activation of ICE1 protein. Furthermore, the regulation of ICE1 can result from multiple post-translational modifications, including ubiquitin-dependent protein degradation, small ubiquitin-related modifier (SUMO) (Barrero-Gil and Salinas, 2013). For instance, HOS1, a RING-finger E3 ubiquitin protein ligase, which can ubiquitinate ICE1 and lead to its proteosomal degradation under cold stress to prevent CBF-mediated expression of COR genes (Dong et al., 2006). By contrast, sumoylation can help stabilize ICE1 by suppressing the ubiquitination, thereby increasing its activity. An excellent example of *in vitro* experiment in *Arabidopsis* indicated that the balance of sumoylation and ubiquitination (such as the system of SIZ1-HOS1) determines ICE1 level (Miura et al., 2007). Here, the significant up-regulation of three E3 SUMO-protein ligases (6.6-fold c45908.graph_c0, 4.0-fold c17726.graph_c0, 2.6-fold c38959.graph_c0) that are highly homologous to SIZ1, and down-regulation of multiple E3 ubiquitin protein ligases supported that they contributed a fine tuning of CBF expression (Supplementary Table S3 in Supplementary Material).

Alternatively, transcription of the CBF is controlled by other regulators via specific *cis*-elements in CBF gene promoters. For example, MYB15 (an R2R3-MYB family protein) can repress the CBF expression by binding to MYB recognition element in CBF promoter during cold treatment (Agarwal et al., 2006). Moreover, some C2H2-type zinc finger proteins such as ZAT12, ZAT10, and AZF2, are also predicted to encode transcriptional repressors of CBF (Sakamoto et al., 2004; Knight and Knight, 2012). Here, multiple MYB and MYB-related TFs and Zinc finger proteins (like ZAT6, ZAT11, and AZF3) were observed and presented different regulation pattern (**Figure 6**; Supplementary Table S3 in Supplementary Material). This pattern is tightly linked with different regulatory motifs in CBF promoters including the MYC- and MYB-recognition elements, and G-box sequences (Maruyama et al., 2012). Moreover, recent researches implicated that the light and circadian signaling pathways are important for the expression of CBF. Some key circadian and phytochrome associated TFs, such as *CIRCADIAN CLOCK ASSOCIATED1* (CCA1), phytochrome-interacting Factor (FIF), phytochrome B (Phy B), regulate cold-inducible expression of CBF and confer responsiveness to circadian gating (Kidokoro et al., 2009; Seo et al., 2012). Consistent with these reports, these genes were captured in our transcriptome data and they played an important role in maintaining a circadian rhythmic regulation for cold-induced transcriptome and minimizing the potentially detrimental effects of sustained CBF expression.

In addition to CBF-dependent pathway, ABA-dependent regulatory system is activated in the response to low temperature and contributes increased cold tolerance. The pathway commonly occurs via ABA-responsive element binding factors (ABFs) encoding bZIP TFs, or MYC and MYB TFs, which interact with ABRE or MYCRE/MYBRE elements in the promoters of cold responsive genes (Maruyama et al., 2012; Jiang et al., 2013). For example, overexpression of *Gm*bZIP44, *Gm*bZIP46, and *Gm*bZIP62 in *Arabidopsis* result in increased freezing tolerance (Liao et al., 2008). Here, many members of the bZIP TF family (29 up-regulation and 27 down-regulation), referred to as ABFs, partly resulted in the increased or decreased expression levels of ABA-synthetic genes (ZEP and NCED3) and ABA-induced genes (RAB18, ABO5, and ASR1) (**Figure 6**). Similarly, the function of MYC/MYB proteins has been found as cooperative activators in the ABA-dependent regulatory pathway (Abe et al., 2003). In line with the finding, a large number of MYC/MYB homologous genes were detected in our transcriptomic data and played a key role in reprogramming ABA dependent gene expression such as COR47, lipid-transfer proteins (LTPs) and LEAs (**Figure 6**).

In summary, the large-scale “omics” technologies utilized here to investigate the overall temporal transciptome and metabolome reprogramming associated with CA are surprisingly informative in uncovering novel networks and hub genes. Consequently, massive transcriptome and metabolome reprogramming occurred during CA in comparison with control. By combining transcriptome and metabolome analyses, we constructed a gene to metabolite network, and found that the CA associated processes are highly energy demanding in DOKM. Furthermore, based on gene interaction and regulation network analysis tools, we found that transcriptional regulators which are persistently induced during CA, can be responsible for maintaining the cold acclimated status vis a comprehensive regulatory mechanism (**Figure 6**). These results can contribute a deeper understanding of the highly complex regulatory program during CA. Some marker COR genes are helpful to enhance cold resistance of DOKM in chilling growth condition by further function analyses.

## Author Contributions

Z-GW designed and performed the experiment and most of the analysis, and drafted the manuscript. WJ helped to analyze the transcriptome data and improve figure quality. NM helped to draft the text and improve English expression. S-LC participated in RNA extraction, gene validation. Z-MT and C-XJ performed cold treatments. All authors read and approved the final manuscript.

## Conflict of Interest Statement

The authors declare that the research was conducted in the absence of any commercial or financial relationships that could be construed as a potential conflict of interest.

## Footnotes

1 https://www.nist.gov/

2 http://bonsai.hgc.jp/mdehoon/software/cluster/software.htm

3 http://jtreeview.sourceforge.net/

4 http://trinityrnaseq.sourceforge.net/

5 http://kobas.cbi.pku.edu.cn/

6 http://cufflinks.cbcb.umd.edu

7 http://www.genome.jp/kegg/
