# Supplementary material for: Insights from the Cold Transcriptome and Metabolome of Dendrobium officinale: Global Reprogramming of Metabolic and Gene Regulation Networks during Cold Acclimation
Source: Front Plant Sci. 2016 Nov 8;7:1653. doi: 10.3389/fpls.2016.01653 (PMC5099257; doi:10.3389/fpls.2016.01653)
Supplement: Supplementary file 1 [file Presentation_1.PDF]

Supplementary Material

Insights from the cold transcriptome and metabolome of *Dendrobium officinale*: global reprogramming of metabolic and gene regulation networks during cold acclimation

Zhi-Gang Wu, Wu Jiang, Song-Lin Chen, Nitin Mantri, Zheng-Ming Tao, Cheng-Xi Jiang

*** Correspondence: Zhi-Gang Wu**，Email：wuzhigang177@126.com

Supplementary Figure S1-S6:

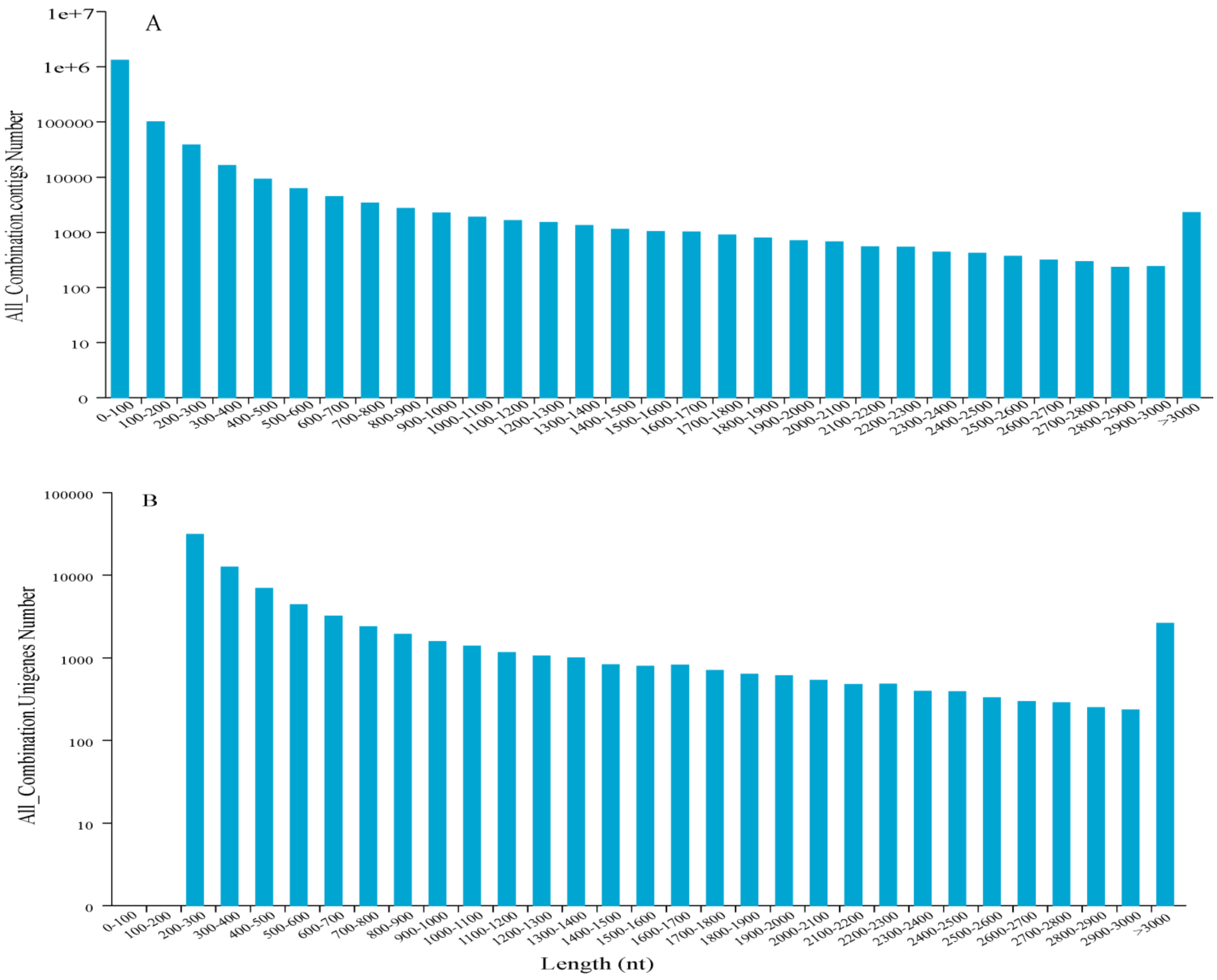

Figure S1. Length distribution of (A) contigs and (B) unigens assembled in *Dendrobium officinale* leaf transcriptome.

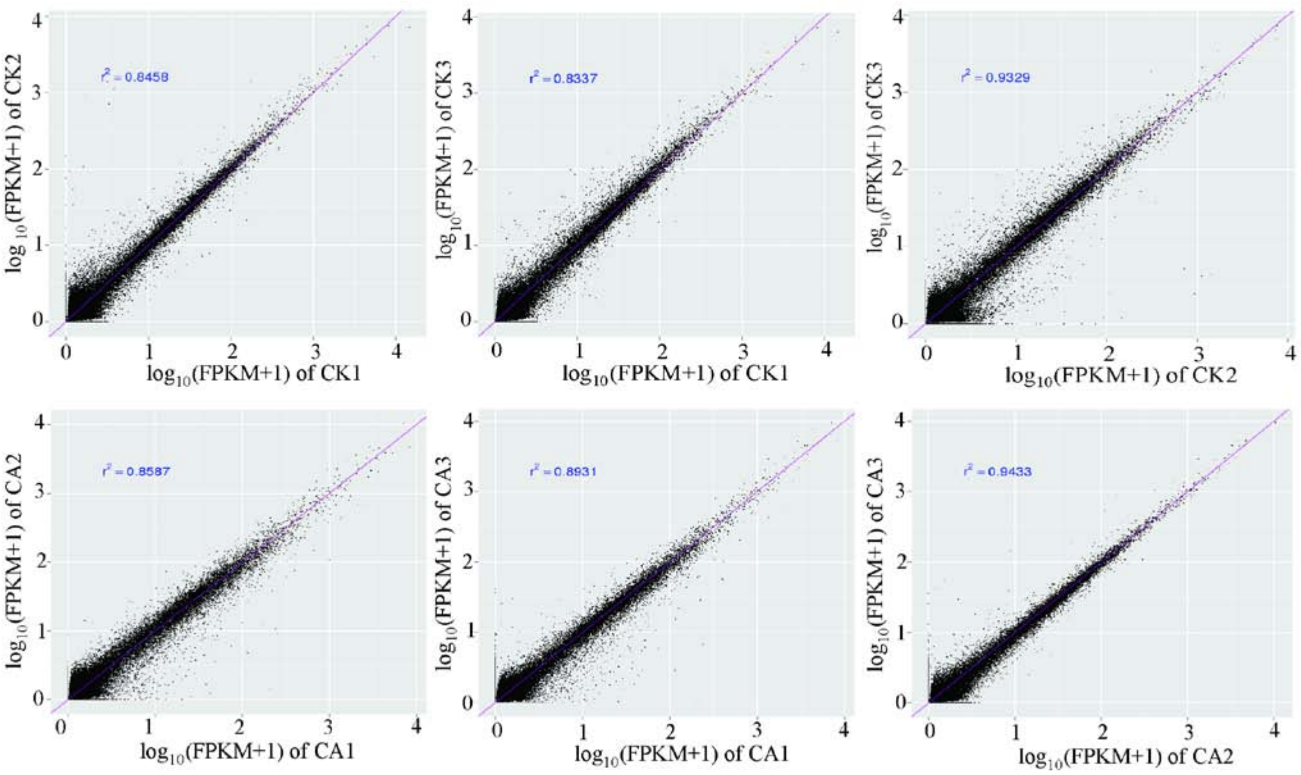

Figure S2. Correlation between biological replicates. For transcriptome analysis, three independent biological replicates for cold acclimation and control treatments were used to construct RNA-seq libraries. The normalized data of $\log_2$ (FPKM value + 1) are highly correlated ($r^2 > 0.83$) between replicates in the same treatments, indicating that all collected samples were well processed.

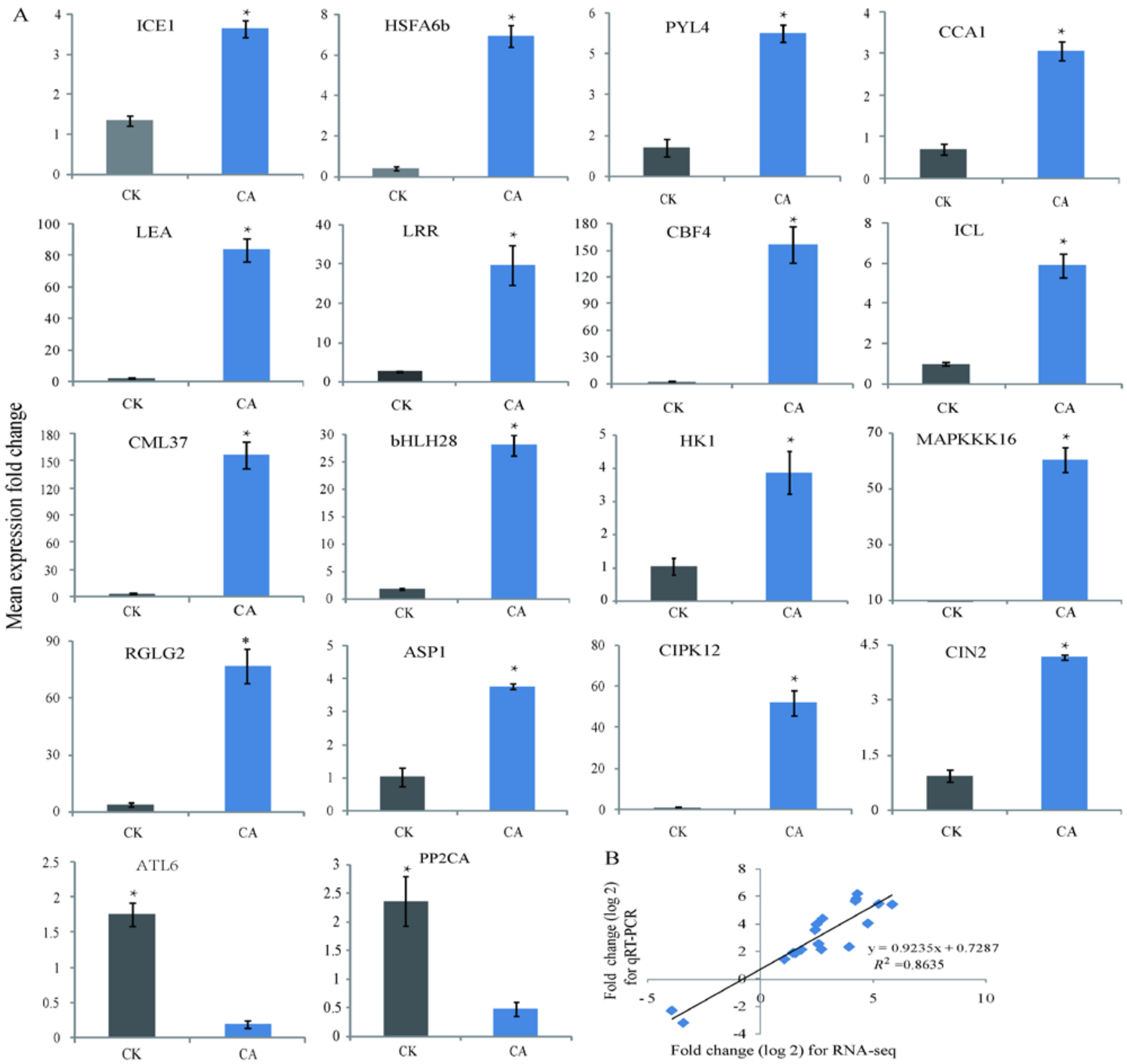

Figure S3. Validations of RNA-seq data of *Dendrobium officinale*. (**A)** Expression profiles for some marker cold-induced genes were validated using qRT-PCR assay. Fold change in gene expression was quantified by the means of three independent qRT-PCR experiments ± SD. Asterisks (*) represent indicates significantly different at $P < 0.01$. (**B)** Scatter plot represents fold change in gene expression measured by RNA-seq and qRT-PCR assay for the selected genes, indicating that the two technologies have a good agreement by calculating its correlation (R^2=0.8635). ATL6, E3 ubiquitin-protein ligase ATL6; ASP1, aspartate aminotransferase 1; CBF4, C-repeat-binding factor 4; CCA1, circadian clock associated 1; CIN2, cell wall invertase 2; CIPK12, CBL-interacting protein kinase 12; CML37, calmodulin like 37; HK1, hexokinase-like 1; HSFA6b, heat shock transcription factor A6B; ICE1, inducer of CBF expression 1; ICL, isocitrate lyase; LEA, Late embryogenesis abundant protein; LRR, Leucine-rich receptor-like protein kinase family; MAPKKK16, mitogen-activated protein kinase kinase kinase 16; PP2CA, protein phosphatase 2CA; PYL4, abscisic acid receptor PYL4-like; RGLG2, E3 ubiquitin-protein ligase RGLG2.

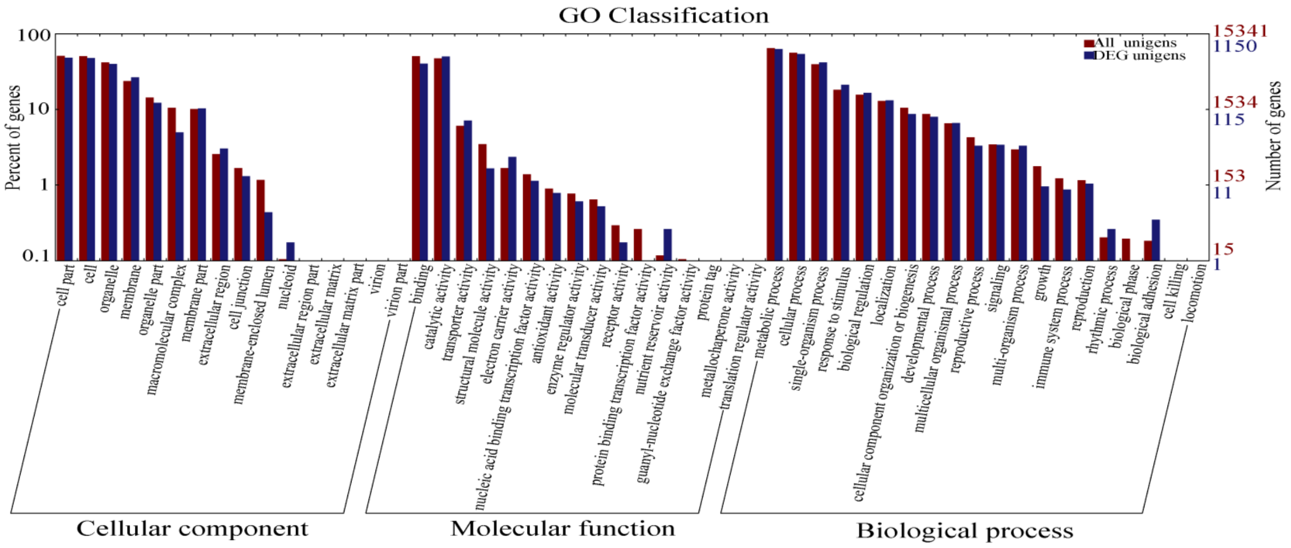

Figure S4. GO functional annotation for all assembled unigenes and DEGs during CA in *Dendrobium officinale*. The numbers at the left and right of the map represent the percentage and amount of genes annotated for each GO category, respectively. A total of 15,341 unigenes and 1,150 DEGs are classified into 52 GO terms: 16 terms in cellular component (GO:0005575), 16 terms in molecular function (GO:0003674) and 20 terms in biological process (GO:0008150).

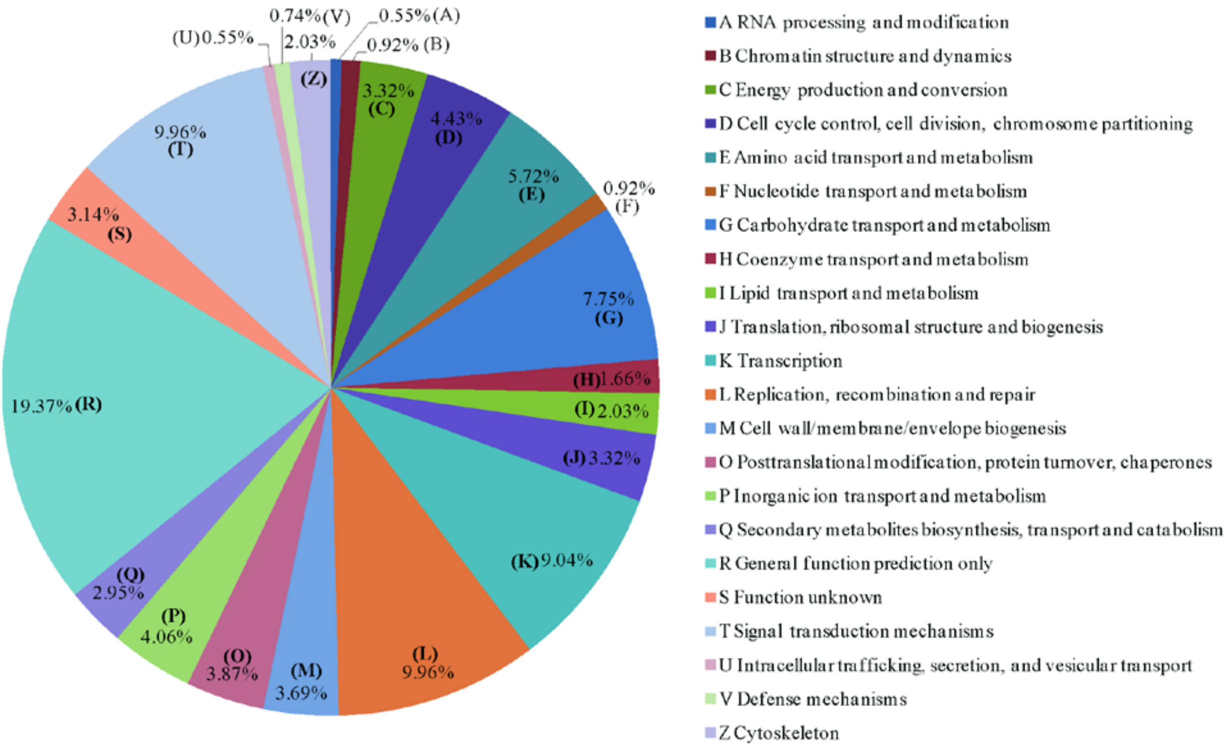

Figure S5. COG functional categories of up-regulated genes during cold acclimation in *Dendrobium officinale*. The percentage of genes annotated for each COG category is shown in the Pie map. A total of 542 up-regulated genes showing significant homology to the COGs database within NCBI (E-value≤1.0 e^{-5}) were classified into 22 categories.

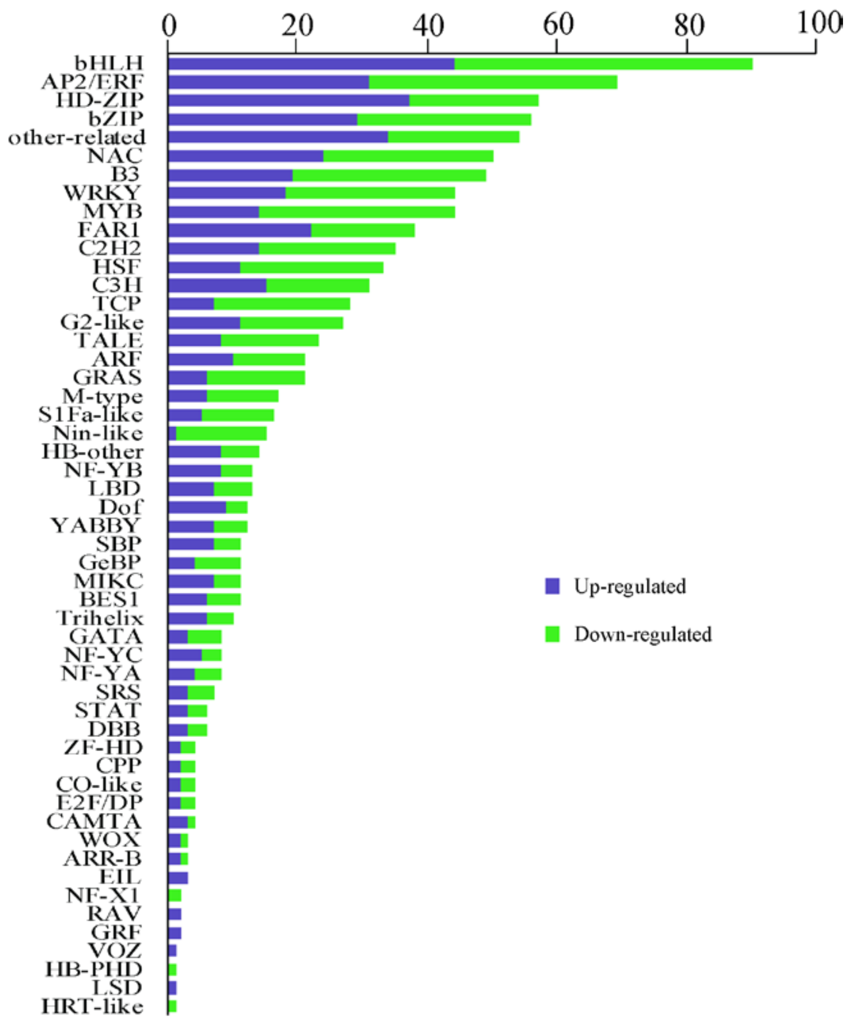

Figure S6. Differentially expressed transcription factors during cold acclimation in *Dendrobium officinale*. A total of 1018 genes belonging to 52 families were differentially expressed during CA as compared with control growth.
